# Racial and ethnic disparities in predictors of glycemia: a moderated mediation analysis of inflammation-related predictors of diabetes in the NHANES 2007–2010

**DOI:** 10.1038/s41387-018-0064-7

**Published:** 2018-10-22

**Authors:** Sarah Nowlin, Charles M. Cleland, Niyati Parekh, Holly Hagan, Gail Melkus

**Affiliations:** 1 Postdoctoral Fellow, New York University Rory Meyers College of Nursing, 433 First Avenue, 7th Floor, New York, NY 10010 USA; 2 New York University Rory Meyers College of Nursing, 433 First Avenue, 7th Floor Room 737, New York, NY 10010 USA; 3 New York University Global Public Health 715-719 Broadway Room 1220, New York, NY 10003 USA; 4 New York University Rory Meyers College of Nursing, 433 First Avenue, 7th Floor Room 752, New York, NY 10010 USA; 5 New York University Rory Meyers College of Nursing, 433 First Avenue, 7th Floor Room 744, New York, NY 10010 USA

## Abstract

**Background/Objective:**

Racial/ethnic disparities in type 2 diabetes (T2D) outcomes exist, and could be explained by nutrition- and inflammation-related differences. The objective of this study is to identify associations between race/ethnicity and glucose control among participants from NHANES 2007–2010, as influenced by diet quality, body mass, and inflammation and grouped by T2D status.

**Subjects/Methods:**

The following is a cross-sectional, secondary data analysis of two NHANES data cycles spanning 2007–2010. The association between race/ethnicity and hemoglobin A1c (HbA1c) as mediated by dietary intake score, body mass index (BMI), and C-reactive protein (CRP) was assessed, as was the strength of the difference of that association, or moderation, by T2D status. The sample included *n* = 7850 non-pregnant adult participants ≥ 20 years of age who had two days of reliable dietary recall data, and no missing data on key variables included in the analysis. The primary outcome examined was HbA1c.

**Results:**

The model accurately explained the variation in HbA1c measures in participants without T2D, as mediated by diet quality, BMI, and CRP. However, significant variation in HbA1c remained after accounting for aforementioned mediators when contrasting non-Hispanic White to non-Hispanic Black participants without T2D. The model was not a good fit for explaining racial/ethnic disparities in HbA1c in participants with T2D. A test of the index of moderated mediation for this model was not significant for the differences in the effect of race/ethnicity on HbA1c by T2D status (moderator).

**Conclusions:**

This study demonstrated that diet quality, BMI, and CRP mediated the effect of race/ethnicity on HbA1c in persons without T2D, but not in persons with T2D. Further research should include additional inflammatory markers, and other inflammation- and T2D-related health outcomes, and their association with racial/ethnic disparities in diabetes.

## Introduction

Racial/ethnic minorities experience worse health outcomes in all-cause mortality, diabetes, cardiovascular disease, and obesity. Inflammation is a common underlying mechanism in the pathophysiological processes of these chronic diseases, which includes type 2 diabetes (T2D). Racial/ethnic minorities with T2D exhibit higher levels of inflammation, as well as poorer glycemic control, than NHW (non-Hispanic Whites) with T2D^1^. There is a dearth of research seeking to explain whether race and ethnicity is a source of disparities in inflammatory and glycemic health outcomes, and whether this could contribute to higher hemoglobin A1c (HbA1c) observed in racial/ethnic minority populations in the United states. Poor diet quality is also a risk factor for T2D and inflammation, and several dietary patterns have been shown to decrease inflammation, particularly those that emphasize high intake of grains, omega-3 fatty polyunsaturated fatty acids and anti-oxidant vitamins^2^. Although dietary intake patterns, inflammation, and T2D are associated, the contribution of these factors to racial/ethnic health disparities in HbA1c is unclear.

**Fig. 1:**
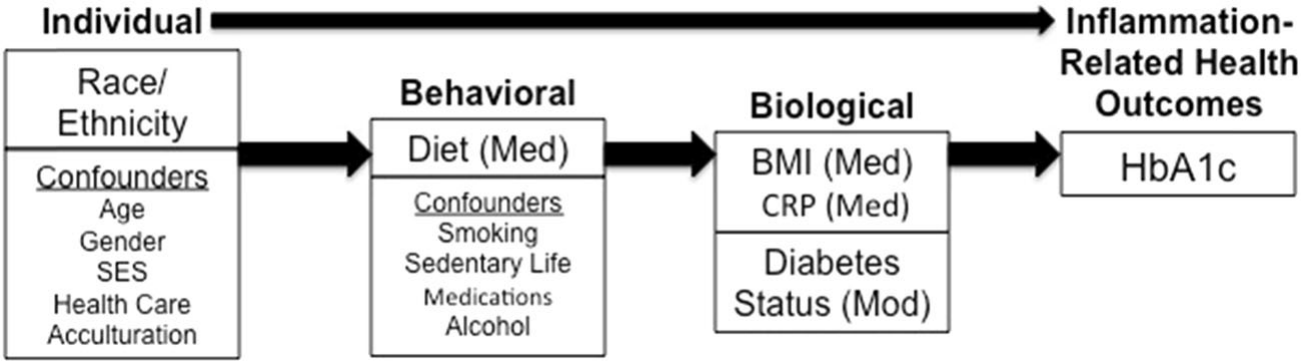
Theoretical model HbA1c- hemoglobin A1c; BMI- body mass index; CRP- C-reactive protein; Med- Mediator; Mod- Moderator. Medications include antihypertensives, antihyperglycemics, aspirin, statins, non-steroidal anti-inflammatory drugs, and estrogen-containing medications; SES- socioeconomic status

Eliminating health disparities is a goal of several national health organizations including Healthy People 2020, and National Institutes of Health. The proposed theoretical model guiding this study is based on two frameworks: the bio-behavioral Framework by Kang et al.^3^, which focuses on stress and inflammation, and the Conceptual Framework model for disparities in endocrine disorders by Golden et al.^4^ (See Fig. 1). The Conceptual framework by Golden et al.^4^ outlines factors that contribute to disparate health outcomes in endocrine disorders, and identifies biological and non-biological factors related to disparities in diabetes health outcomes. The bio-behavioral Framework by Kang and colleagues is a framework meant to guide research in the area of stress, inflammation, and health^3^. This framework identifies inflammation as a biological factor related to the immune system that influences health outcomes^3^. Merging the aforementioned frameworks;^3,4^ Fig. 1 illustrates the association of individual, psychosocial, behavioral, biological, and environmental factors influencing health outcomes. The aim of this study is to use structural equation modeling (SEM) to identify how race/ethnicity influences HbA1c through the proceeding mediating factors, which lie on the causal pathway: dietary pattern, body mass index (BMI), and C-reactive protein (CRP). T2D status is hypothesized to moderate the influence of race/ethnicity on HbA1c, which would cause a change in the strength of the association between the two variables^5^.

## Methods

### Design

The National Health and Nutrition Examination Survey (NHANES) study is a continuous nationwide survey conducted by the Centers for Disease Control and Prevention with a goal of describing the “health and nutrition status of the adults and children of the United States”^6^. NHANES uses multi-stage probability cluster sampling of non-institutionalized persons to systematically collect data from a representative sample of the US population^6^. After an in-home survey to determine eligibility, participants travel to the local mobile examination center (MEC) to have a physical exam, blood draw, and to provide their first 24-hour dietary recall (24 h). Within 3–10 days, a second 24 h is conducted over the telephone. Data from both 24 h were used in this study.

### Population/sample

All NHANES data have been de-identified and each participant assigned a sequence number. This cross-sectional, secondary data analysis will use data from two collection cycles of the NHANES study over the years 2007–2010, as these were the most recent cycles that included CRP measurements. Each data collection cycle lasts 12 months^6^. The survey over-samples non-Hispanic Black (NHB) and all Hispanic subgroups to obtain a sufficient, representative sample of non-institutionalized U.S. citizens in those racial/ethnic groups^6^.

This secondary data analysis had a specific focus on the adults who self-identified as (1) NHW, (2) NHB or African American, and (3) Hispanic Mexican American or another Hispanic group. The NHANES database does contain a fourth category for race/ethnicity for participants who reported other racial/ethnic groups, but owing to the heterogeneity of this group and its small sample size, it was not included in this analysis.

The following inclusion criteria were used to select the sample for this secondary data analysis: (1) complete data from the interview and MEC exam; (2) was ≥ 20 years of age at the time of the interview; (3) identified race/ethnicity as either NHW, NHB, Mexican American, or other Hispanic; and (4) complete dietary data from 24 h. Exclusion criteria for selecting participants for this secondary analysis included pregnancy, absence of complete or reliable 24 h, or were determined by a systematic approach described below, to have type 1 diabetes (T1D) (*n* *=* 32). Subjects with missing data on any covariate or main outcome variable were also excluded (*n* *=* 1332). After applying these criteria to the 2007–2010 NHANES data set, a total of *n* *=* 7850 subjects remained.

### Variables

#### Exposure variable

Race/ethnicity data were self-reported by the participant and categorized into NHW, NHB, and Hispanic.

#### Outcome variable

The primary outcome variable for this secondary data analysis is glycohemoglobin % (HbA1c), which is a measure of glucose control over the past 8–12 weeks. All serum samples were collected on one occurrence in the MEC provided by NHANES. All laboratory procedures have been discussed at length in publications describing the safety, reliability, and validity of the procedures used to handle specimens^7^.

### Mediating variables

**Fig. 2:**
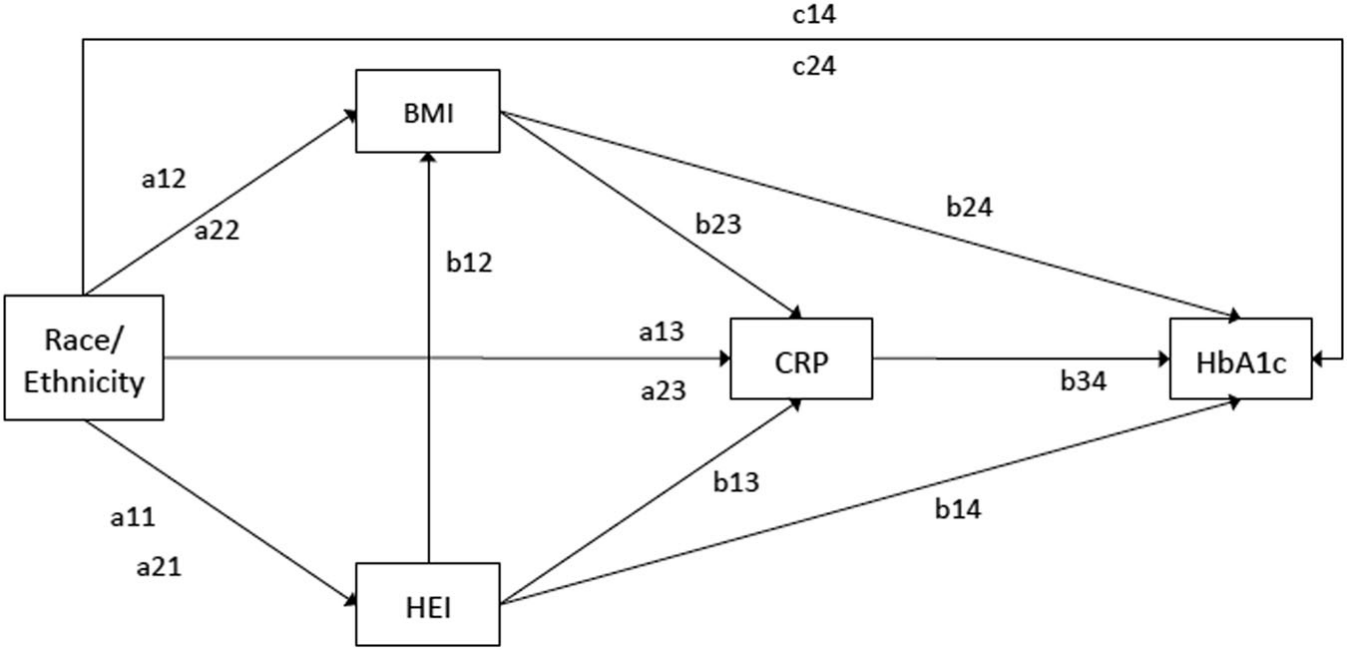
Multiple mediation path model The path model depicts the relationship between race/ethnicity and HbA1c, as mediated by HEI, BMI, and CRP. Paired ‘a’ and ‘c’ paths represent a contrast of each minority group (Hispanic or NHB) to NHW. All paths listed are hypothesized to be moderated by diabetes status. The total indirect effect of the model is tested for moderated mediation index introduced by Hayes, 2015

In this SEM, the effect of race/ethnicity on HbA1c transmits through three mediating variables (Healthy Eating Index–2010 score (HEI), BMI, and CRP), shown in Fig. 2. Mediation implies a process or chain of effects, in this case, with racial and ethnic disparities in HbA1c expected to arise by means of indirect effects through HEI, BMI, and CRP.

In other words, indirect or mediated effects are constituted by a multiplicative chain of regression coefficients. Dietary scores for the HEI were created using the SAS code available on the USDA website. This analysis did not utilize the Monte Carlo simulation suggested in the original SAS code, as this would inhibit the use of unique indicators in further data analysis. Each participant who completed the first 24 h was asked to complete a second. Means of the two total scores were created and used as a measure of diet quality. The HEI is a continuous variable with a range of 0 to 100, with a score of 100 indicating perfect adherence to the USDA dietary guidelines.

BMI is a variable in the NHANES database, and is used as a proxy measure of adiposity in the present study. Weight in kilograms is divided by height (in meters squared), which were measurements taken at the MEC exam. Serum samples from participants assessed presence of chronic inflammation (CRP), HbA1c, and fasting plasma glucose in the selected sample. The CRP samples were analyzed using latex-enhanced nephelometry with a high-sensitivity CRP reagent, which should allow detection of the lower CRP levels associated with chronic low-grade inflammation^8^.

### Covariates

Covariates include age, gender, time spent in the United States, poverty index ratio (PIR), physical activity, and smoking^9^. Time spent in the United States was used as a proxy measure of acculturation. PIR is a ratio of family income to the poverty level, and is calculated by NHANES. Physical activity was measured by a self-reported (yes/no) response to the following question: “Do you do any vigorous-intensity sports, fitness, or recreational activities that cause large increases in breathing or heart rate like running or basketball for at least 10 minutes continuously?” Smoking was recoded, to reflect categories used in the literature, as “not at all”, “some days”, and “every day”. Use of the following medications (yes/no) were also considered as they have been shown in previous research to impact glucose metabolism and/or inflammation: HMG-CoA reductase inhibitors (statins), aspirin, oral anti-diabetic medications, insulin, non-steroidal anti-inflammatory drugs, steroids, and medications that alter systemic estrogenic activity.

### Moderating variable

**Fig. 3:**
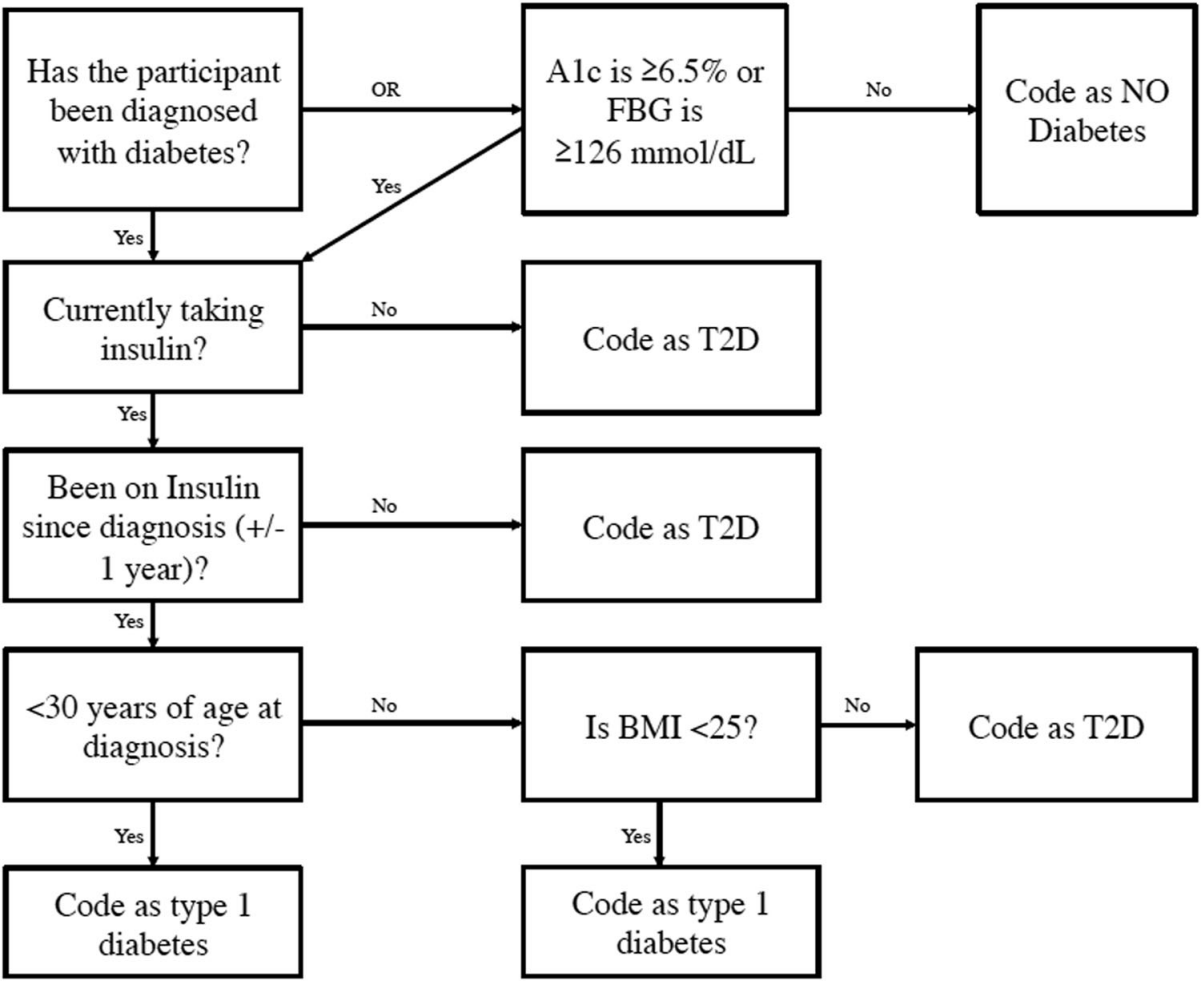
Flowchart of identification of participants from NHANES 2007–2010 with T2D A1c- hemoglobin A1c; BMI- body mass index; FBG- fasting blood glucose; T2D- type 2 diabetes

T2D status was the moderating variable in the SEM, and was coded as yes/no. Moderation implies interaction effects, but also puts the focus of stratified or simple main effects on the exposure of interest (race/ethnicity), conditional on the moderator (T2D). Figure 3 displays the systematic approach used to detect participants with T2D, and to exclude participants with T1D (*n* = 27), as the format of the question concerning diabetes status in NHANES is non-specific regarding diabetes type.

### Data analysis

Descriptive statistics were calculated using SPSS version 22, using the complex samples module. Unweighted counts of participants were noted per racial/ethnic group, and are presented as percentages for categorical variables, or means ( ± SE) for continuous variables. CRP measures were log-transformed to reduce positive skew. BMI was treated as a categorical variable for descriptive purposes, but treated as a continuous variable in the regression models. Moderated mediation Model 5 discussed in Preacher, Rucker, & Hayes was used to guide the SEM plan^10^. This moderated mediation model tests the hypothesis that associations between race/ethnicity and the three mediators (BMI, CRP, and HEI), as well as associations between the three mediators and the outcome variable (HbA1c) are conditional on, or moderated by, T2D status^10^. The index and test of the dichotomous moderator (T2D) suggested by Hayes^11^ was also used to test the moderation of indirect effects from race/ethnicity to HbA1c by T2D status.

Analyses in the moderated multiple mediation model were conducted in Mplus Software version 7. Mediators (HEI, BMI, and CRP) and the outcome measure (HbA1c) were treated as continuous variables. Standard errors were estimated by the replicate weights methodology using bootstrapping at the PSU level of the complex sample design, with 10,000 bootstrap draws. Each indirect path from race/ethnicity to HbA1c was assessed using the product of coefficients strategy; all unstandardized coefficients (B), standard errors, and 99% confidence intervals (CI) are reported. Although model fit is typically reported for SEM analyses, the structural portion of this moderated mediation model is saturated, and therefore the focus is on estimated rather than fixed parameters.

The path model in Fig. 2 depicts the relationship between race/ethnicity and HbA1c, as mediated by HEI, BMI, and CRP. The ‘A’ paths represent direct effects from race/ethnicity to HEI, BMI, CRP, and HbA1c. The ‘B’ paths represent the direct effects from the mediators either to other mediators or to HbA1c. Path ‘C’ represents the direct path from race/ethnicity to HbA1c, and signifies the variance in the association between race/ethnicity and HbA1c not explained by HEI, BMI, or CRP.

## Results

This analysis included *N* *=* 7850 adult participants from the 2007–2010 cycles of NHANES who completed two 24 h and had no missing data for the selected outcome variables. There were *n* *=* 6562 participants without T2D, and *n* *=* 1288 participants in the T2D group. Findings were considered statistically significant if the 99% CI did not include zero.

Table 1 presents study characteristics by race/ethnicity, showing percentages, and for continuous variables, the mean and standard error. For all racial/ethnic groups, the oldest group had the lowest proportion of participants, with between 72–80% falling in the 20–59 age range. About half of the participants were male, with the majority of all participants being non-smokers. Hispanics represented the largest proportion with only a high school education or below (37%), whereas NHW participants had the highest proportion with a college education or beyond (62%). Almost half of the Hispanic participants were below the poverty threshold, as compared with only 15% of NHW. The proportion of persons not born in the United States was highest among Hispanics (59.2%). HEI scores ranged from 14 to 98, with an unadjusted mean of 53.5 for all participants. The adjusted means for those with and without diabetes were 55.7 and 58.8, adjusted for all covariates.

**Table 1:** Characteristics of the population and present means, standard errors by race/ethnicity

	All	NHW	NHB	Hispanic
	*n* *=* 7850	*n* *=* 4247	*n* *=* 1461	*n* *=* 2142
Age years				
20–39	35.8 (1.0)	32.0 (1.2)	41.2 (2.4)	53.1 (1.9)
40–59	39.7 (0.7)	40.5 (0.9)	40.2 (1.9)	34.4 (1.4)
≥ 60	24.5 (0.8)	27.5 (0.9)	18.6 (1.1)	12.5 (1.1)
Sex % male	47.4 (0.6)	47.3 (0.8)	43.7 (1.7)	50.8 (1.2)
Physical activity				
Yes	24.0 (1.4)	24.2 (1.8)	24.6 (1.9)	22.1 (1.3)
Education				
HS or below	42.5 (1.7)	38.0 (2.5)	49.3 (1.5)	63.1 (2.2)
Beyond	57.5 (1.7)	62.0 (2.5)	50.7 (1.5)	36.9 (2.2)
Poverty index ratio %				
< 1.29	21.0 (1.2)	15.0 (1.6)	32.5 (2.2)	45.6 (1.9)
1.3–3.49	34.8 (1.2)	33.4 (1.5)	42.4 (1.9)	36.5 (1.7)
≥ 3.5	44.2 (1.6)	51.6 (2.3)	25.1 (2.0)	17.9 (1.4)
Diabetes status				
T2D	11.1 (0.6)	9.9 (0.8)	17 (1.0)	13.2 (1.0)
HEI total score (mean, SE)	53.5 (0.5)	54.0 (0.6)	50.0 (0.7)	53.4 (0.5)
Smoking status				
Not at all	79.4 (1.0)	79.9 (1.3)	73.2 (1.9)	81.7 (1.5)
Some days	3.4 (0.3)	2.6 (0.3)	5.3 (0.8)	5.8 (0.7)
Every day	17.3 (1.0)	17.5 (1.3)	21.5 (1.9)	12.5 (1.3)
BMI (mean, SE)	28.9 (0.1)	28.6 (0.2)	31.0 (0.3)	29.4 (0.2)
HbA1c (mean, SE)	5.6 (0.0)	5.5 (0.0)	5.8 (0.0)	5.6 (0.0)
CRP (mean, SE)	0.39 (0.0)	0.37 (0.0)	0.56 (0.0)	0.41 (0.0)
Time in United States				
< 5 years	1.4 (0.2)	0.6 (0.2)	1.3 (0.4)	6.6 (1.0)
5–19.99 yr	5.5 (0.7)	1.4 (0.4)	4.2 (0.9)	29.5 (2.7)
> 20 years	5.5 (0.6)	2.7 (0.4)	3.6 (1.5)	23.1 (1.5)
Born in United States	87.6 (1.3)	95.3 (0.7)	91.0 (2.1)	40.8 (2.9)

*T2D* type 2 diabetes, *HEI* Healthy Eating Index-2010, *BMI* body mass index, *CRP C*-reactive protein, *HbA1c* hemoglobin A1c, *HS* high school, *NHW* non-Hispanic White, *NHB* non-Hispanic Black

### Direct effects

As presented in Table 2, in the group without T2D, NHB had lower HEI scores than NHW participants (a21: *B* = −2.56; (−4.37, −0.70)), but the difference between Hispanic and NHW participants was not significant (a11: *B* = −0.70; (−2.65, 1.41)). In terms of the direct effect, among those without T2D, HbA1c was significantly higher in NHB compared with NHW (c24: B = 0.11; (0.06, 0.17)).

**Table 2:** Regression coefficients and 99% confidence intervals for direct effects in the path model

		No T2D		T2D present	
		*B*	99% CI	*B*	99% CI
HEI regressed on					
a11	Hispanic vs. NHW	−0.701	−2.65, 1.408)	−2.301	(−5.626, 1.942)
a21	NHB vs. NHW	−2.564	(−4.371, −0.698)	−1.015	(−3.564, 2.122)
BMI regressed on					
a12	Hispanic vs. NHW	1.006	(0.201, 1.807)	-0.080	(−2.264, 2.483)
a22	NHB vs. NHW	1.987	(1.170, 2.765)	0.847	(−1.497, 3.151)
b12	HEI	−0.044	(−0.064, −0.022)	−0.038	(−0.094, 0.016)
CRP regressed on					
a13	Hispanic vs. NHW	0.103	(0.037, 0.166)	0.109	(−0.053, 0.223)
a23	NHB vs. NHW	0.058	(−0.005, 0.123)	0.083	(−0.043, 0.213)
b13	HEI	−0.002	(−0.003, 0.000)	−0.003	(−0.006, 0.001)
b23	BMI	0.041	(0.038, 0.044)	0.025	(0.019, 0.032)
HbA1c regressed on					
c14	Hispanic vs. NHW	0.023	(−0.029, 0.073)	0.294	(−0.243, 0.683)
c24	NHB vs. NHW	0.113	(0.059, 0.168)	0.346	(−0.115, 0.779)
b14	HEI	0.000	(−0.001, 0.001)	−0.004	(−0.014, 0.007)
b24	BMI	0.008	(0.005, 0.011)	−0.011	(−0.035, 0.008)
b34	CRP	0.060	(0.025, 0.097)	0.439	(0.171, 0.725)

A 99% CI that does not include zero indicates significance. *T2D* type 2 diabetes, *HEI* Healthy Eating Index−2010, *BMI* body mass index, *CRP* C-reactive protein, *HbA1c* hemoglobin A1c, *NHW* non-Hispanic White, *NHB* non-Hispanic Black, *CI* confidence interval

Coefficients for the direct effects of race/ethnicity on BMI indicate significantly higher BMI among NHB (2 kg/m^2^ higher, *p* < 0.01) and Hispanics (1 kg/m^2^ higher, *p* < 0.01) when compared with NHW in those without T2D. There was no significant difference in BMI by race/ethnicity in those with T2D.

In those with T2D, there was no significant difference in HbA1c levels when comparing NHW to either minority group, keeping constant the mediating effects of HEI, BMI, and CRP. The only significant direct effects in the analysis of those with T2D were BMI on CRP (b23: *B* = 0.03, (0.02, 0.03)), and CRP on HbA1c (b34: *B* = 0.44, (0.17, 0.73)).

### Mediation

Table 3 displays the coefficients and 99% confidence intervals of the specific indirect effects of the mediation model, stratified by T2D status. In persons without T2D, the specific indirect effect of race/ethnicity on HbA1c, through HEI, BMI, and CRP, was significant for Hispanic and NHB persons, when compared with NHW (*B* = 0.00, *p* *<* 0.01; *B* = 0.00, *p* < 0.01, respectively). Keeping the mediating effects of BMI and CRP constant, the specific indirect effect of race/ethnicity on HbA1c through HEI was not significant when comparing NHW with Hispanics and NHB (*B* = 0.00, (−0.001, 0.002); *B* = 0.00, (−0.003, 0.004), respectively). All other specific indirect effects in the group without T2D were significant for an effect of race/ethnicity on HbA1c (data not shown). For those with T2D, none of the coefficients for the specific indirect effects of race/ethnicity on HbA1c through the proposed mediators were significant.

**Table 3:** Indirect effects of the association of race/ethnicity on HbA1c, mediated by HEI, BMI, and CRP, stratified by T2D status

Specific indirect effect paths	No T2D		T2D present	
	*B*	99% CI	*B*	99% CI
R/E → BMI → HbA1c				
Hispanic vs. NHW	0.008	(0.001, 0.018)	0.001	(−0.054, 0.042)
NHB vs. NHW	0.016	(0.008, 0.026)	−0.010	(−0.067, 0.025)
R/E → HEI → HbA1c				
Hispanic vs. NHW	0.000	(−0.001, 0.002)	0.009	(−0.022, 0.044)
NHB vs. NHW	0.000	(−0.003, 0.004)	0.004	(−0.013, 0.028)
R/E → CRP → HbA1c				
Hispanic vs. NHW	0.006	(0.001, 0.014)	0.048	(−0.023, 0.127)
NHB vs. NHW	0.004	(0.000, 0.010)	0.036	(−0.015, 0.122)
R/E → HEI → BMI → CRP → HbA1c				
Hispanic vs. NHW	0.000	(0.000, 0.000)	0.001	(−0.003, 0.007)
NHB vs. NHW	0.000	(0.000, 0.001)	0.000	(−0.001, 0.003)
Total indirect effect				
Hispanic vs NHW	0.026	(0.016, 0.038)	0.041	(−0.023, 0.126)
NHB vs NHW	0.017	(0.007, 0.032)	0.060	(−0.026, 0.137)
Index & Test of Moderated Mediation				
Hispanic vs. NHW		−0.043 (−0.107, 0.041)		
NHB vs. NHW		−0.016 (−0.073, 0.045)		

*R/E* Race/Ethnicity, *HEI* Healthy Eating Index, *BMI* Body Mass Index, *CRP* C-reactive protein, *NHB* non-Hispanic Black, *NHW* non-Hispanic White; *T2D* type 2 diabetes. Findings were considered significant if the 99% CI does not include zero

Taking into account the effect of HEI on BMI, and BMI on CRP levels, the total indirect effect of race/ethnicity through HEI, BMI and CRP on HbA1c was significant, but weak, when comparing NHW with NHB and Hispanic participants without T2D (*B* = 0.02; (0.01, 0.03), and *B* = 0.03; (0.02, 0.04), respectively). Total indirect effects for these paths were not significant for participants with T2D.

### Standardized effects

The mediation model was rerun with HEI, BMI, CRP, and HbA1c variables standardized for alternative estimates of effect sizes in each T2D group. Standardized mean differences in HEI, BMI, CRP, and HbA1c by race/ethnicity (d) and standardized regression coefficients (*β*) for direct effects of continuous variables are presented in Table 4. Effect sizes for race/ethnicity contrasts on HEI, BMI, CRP, and HbA1c were small in those without T2D. However, there was a moderate direct effect of BMI on CRP (*β* = 0.470; (0.434, 0.509)), suggesting a 1-SD increase in BMI was associated with about a ½-SD increase in CRP. This effect size was smaller in those with T2D, but significant (*β* = 0.350; (0.274, 0.430)). The expected increase in HbA1c with a 1-SD increase in CRP was small, but significant for those without T2D (*β* = 0.088; (0.037, 0.144)), and slightly larger and still significant in those with T2D (*β* = 0.157; (0.073, 0.249)).

**Table 4:** Standardized effect sizes and 99% confidence intervals for direct effects in the path model, stratified by T2D status

		No T2D		T2D present	
		*β*	99% CI	*β*	99% CI
HEI regressed on					
a11	Hispanic vs. NHW (d)	−0.049	(−0.184, 0.097)	−0.187	(−0.443, 0.128)
a21	NHB vs. NHW (d)	−0.178	(−0.305, −0.047)	−0.108	(−0.300, 0.126)
BMI regressed on					
a12	Hispanic vs. NHW (d)	0.158	(0.031, 0.283)	−0.009	(−0.310, 0.351)
a22	NHB vs. NHW (d)	0.314	(0.185, 0.438)	0.142	(−0.168, 0.433)
b12	HEI	−0.099	(−0.145, −0.051)	−0.062	(−0.164, 0.029)
CRP regressed on					
a13	Hispanic vs. NHW (d)	0.187	(0.065, 0.301)	0.219	(−0.103, 0.440)
a23	NHB vs. NHW (d)	0.108	(−0.010, 0.228)	0.149	(−0.094, 0.423)
b13	HEI	−0.044	(−0.087, −0.001)	−0.075	(−0.167, 0.019)
b23	BMI	0.47	(0.434, 0.509)	0.35	(0.274, 0.430)
HbA1c regressed on					
c14	Hispanic vs. NHW (d)	0.06	(−0.076, 0.193)	0.191	(−0.185, 0.468)
c24	NHB vs. NHW (d)	0.302	(0.157, 0.450)	0.254	(−0.042, 0.535)
b14	HEI	−0.004	(−0.049, 0.038)	−0.046	(−0.124, 0.041)
b24	BMI	0.134	(0.081, 0.193)	−0.056	(−0.168, 0.035)
b34	CRP	0.088	(0.037, 0.144)	0.157	(0.073, 0.249)

β for contrasts of race/ethnicity groups can be interpreted as standardized mean differences (d). *HEI* Healthy Eating Index; *BMI* body mass index; *CRP* C-reactive protein; *NHB* non-Hispanic Black, *NHW* non-Hispanic White, *T2D* type 2 diabetes

### Moderation

Moderation of the multiple mediation model by T2D status was assessed by calculating the index of moderated mediation suggested by Hayes^11^. This is essentially a test of the interaction between T2D and the indirect effect race/ethnicity on HbA1c. The index of moderated mediation with a dichotomous moderator is defined as the difference in the indirect effects, or mediated effects, between the two levels of the moderator, which are T2D status yes or no. The test of this index is assessed by generating a bootstrap 99% confidence interval of the difference in indirect effects across T2D groups, and the result is presented in Table 3. The index and test of moderated mediation was performed for the total indirect effect and was not significant for a moderating effect of T2D on the effect of race/ethnicity on HbA1c through the proposed mediators.

## Discussion

This analysis sought to determine whether the mechanisms that cause racial/ethnic disparities in glycemic control outcomes, as measured by HbA1c, are conditional on T2D status. Results support the bio-behavioral theoretical framework chosen to guide the analysis, as individual (race/ethnicity and the aforementioned covariates included in all regression analyses), behavioral, and biological factors were significantly associated with the studied health outcome. Although the coefficients for the specific indirect effects in this model were significant for an effect of NHB and Hispanic race/ethnicity on HbA1c through the mediators compared with their NHW counterparts in persons without T2D, the effect sizes were very small. However, this analysis does show that the association between race/ethnicity and HbA1c is at least partially mediated by HEI, BMI, and CRP.

Results of the index and test of moderated mediation suggest that T2D does not moderate the effect of race/ethnicity on glycemic control, as measured by HbA1c. Therefore, we could not disprove the null hypothesis. There were, however, several pathways that were, although not significantly, different between the two groups. There were stronger associations between direct effects of race/ethnicity (NHB vs. NHW) on BMI in those without T2D than those with T2D, indicating a greater racial/ethnic disparity in BMI in those without T2D. These associations were not significant when comparing Hispanics to NHW. Similar results were found in a study examining the prevalence of T2D in NHB and NHW at a similar SES. Results from that study showed that for participants who had ever experienced obesity, no racial/ethnic disparity existed in those with T2D^12^. Diet and lifestyle changes that are made with a diagnosis of T2D, as well as controlling for known predictors of T2D prevalence (e.g., age, education, exercise), attenuates the association between race/ethnicity, HEI, and BMI, and limits presence of racial/ethnic disparities in those with T2D.

The weak association between diet quality, as measured by HEI-2010 scores, and CRP in persons with and without T2D in this study was unexpected. Previous research indicates a significant effect of diet on inflammatory markers in both intervention studies as well as cross-sectional studies. Similarly, there was evidence that higher HEI scores are associated with lower BMI in the no T2D group, but this association is not significant in the group with T2D. Chen et al.^13^, were also unable to demonstrate an association between total HEI score and BMI, but showed that higher fruit and vegetable intake was associated with a BMI ≤ 25 kg/m^2^ ^13^. Chen et al. also noted that persons with T2D had higher HEI and lower sugar intake than participants without T2D, which may attenuate the association between HEI and BMI in participants with T2D^13^. Another recent study of NHANES participants found that there was not a racial/ethnic disparity in total HEI score in those with T2D^14^. Therefore, component scores of the HEI may be more revealing of specific food groups that contribute to racial/ethnic disparities in HEI score, CRP, and BMI.

Furthermore, direct effects from race/ethnicity to HbA1c in those with T2D were not significant. This finding indicates that over and above the variability in HbA1c explained by the mediators and covariates included in the model, there is no significant difference in HbA1c by race/ethnicity. Standardized mean differences were also smaller in the T2D group when comparing NHW with NHB. This result is in contrast to a meta-analysis of studies reporting HbA1c levels in persons with T2D that did find significantly higher levels in NHB participants compared with NHW^15^. It is possible that controlling for HEI, BMI, and CRP in the T2D group explained some of the variability in HbA1c observed in these other studies. Previous research has indicated that in T2D, BMI is a strong predictor of CRP, but that association could be different among men and women^16^. This study confirmed that association, in that the direct effect of BMI on CRP was small to moderate and significant in both T2D groups, after controlling for the effect of HEI. Further research into the mechanisms behind differences in these associations by sex should be conducted, as hormonal influences may play a role in fat deposition and inflammation^16^.

Appropriately, the effect of BMI on HbA1c was significant only through the mediating effect of CRP. Similar results were seen in the Action to Control Cardiovascular Risk in Diabetes (ACCORD) trial, in which results for the participants with tight glycemic control demonstrated a significant decrease in CRP, which was more robust after adjusting for changes in BMI over the intervention period^17^. This relationship was completely attenuated after controlling for changes in HbA1c. The ACCORD study highlighted the occurrence of weight gain as a result of tight glycemic control in T2D, which in turn may blunt the observed effect of race/ethnicity on HbA1c in the present analysis. As higher BMI is associated with higher levels of CRP, if this occurs in conjunction with treatment with insulin or another T2D treatment that promotes fat storage, A1c may not be higher (as would be expected).

This model does not demonstrate causation, nor does it suggest the order of events leading to changes in HbA1c levels. Results from this analysis do suggest, however, that there is an association between race/ethnicity and HbA1c that operates through systemic inflammation (CRP) and body mass/fat (BMI). The influence of diet quality on this pathway is weaker comparing NHW with Hispanics than when comparing NHW with NHB, and does not reach significance. The results of this analysis are consistent with previous research in racial/ethnic health disparities. National health statistics suggest that NHB and Hispanics have a much higher rate of obesity than NHW^18^. As greater fat mass leads to macrophage infiltration and increased production of inflammatory cytokines, glucose metabolism is impaired, resulting in higher HbA1c levels.

Some important limitations in this study should be noted. First, as this analysis used cross-sectional NHANES data, it is not possible to establish causation between predictor, covariate, and outcome variables. In addition, inclusion of more indicators of low-grade inflammation may have influenced results. For example, research has shown that IL-6 and TNF-α are associated with the inflammation present in those with T2D. Use of CRP alone may not have captured presence of inflammation, although it is an accepted measure of low-grade inflammation^19^. In addition, in NHANES, data for several variables included in this analysis were collected via self-report, including the 24 h, race/ethnicity, and T2D status, which are three key variables in the analysis. Particularly in recalling dietary data, which relies on memory and is subject to social bias, self-report can lead to measurement error that has not been accounted for in this analysis. Although T2D status was self-reported and non-specific with regards to diabetes type 1 or 2, we believe that pairing self-report data with biological results increases the likelihood that T2D status was assigned appropriately.

Despite these limitations, the strengths of this study should also be noted. SEM allows simultaneous regression of multiple pathways involved in a complex disease process such as that present in T2D. Bootstrapping is also capable of producing accurate confidence intervals without the assumption of normally distributed data, such as that present when using the product of coefficients strategy. Finally, these findings are novel in that previous research seeking to explain racial/ethnic disparities in T2D health outcomes has not used a bio-behavioral framework to guide research, which includes behavioral and biological factors and may provide a more accurate portrayal of the pathophysiological process in T2D.

In conclusion, results from this analysis indicate that HEI, BMI, and CRP do not mediate the association between race/ethnicity and HbA1c once a diagnosis of T2D is established. As previously mentioned, weight gain that can occur with certain treatments for hyperglycemia, whereas reducing HbA1c, can increase weight and cause CRP to increase as well. This phenomenon could be the explanation for the lack of a significant effect of race/ethnicity on HbA1c through the proposed mediation pathways and warrants further analysis through prospective or interventional research. Finally, the bio-behavioral health disparities framework introduced in this paper may successfully explain racial/ethnic health disparities in other T2D-related health outcomes such as renal failure, diabetic retinopathy, and neuropathy^20–22^. Further research into these health outcomes is also warranted.
